# Unveiling a Unique Case of Scimitar Syndrome: Clinical Significance and Multidisciplinary Management Challenges in Pakistan

**DOI:** 10.7759/cureus.53874

**Published:** 2024-02-08

**Authors:** Gulalay Shamal, Anam Israr Khan, Ahsan Ali, Neha James, Moula Ghulam

**Affiliations:** 1 Internal Medicine, Rehman Medical Institute, Peshawar, PAK; 2 General Medicine, Rehman Medical Institute, Peshawar, PAK; 3 Medicine, Rehman Medical Institute, Peshawar, PAK

**Keywords:** abnormal pulmonary vein, adult congenital heart disease (achd), adult scimitar syndrome, congenital heart anomaly, scimitar syndrome

## Abstract

Scimitar syndrome, a rare congenital cardiac anomaly, involves abnormal pulmonary vein drainage into systemic veins, leading to distinct imaging features resembling a curved-blade sword. This case report presents a unique instance of scimitar syndrome in Pakistan, emphasizing its clinical importance and the challenges of management. A 26-year-old female with a history of recurrent pulmonary infections and respiratory symptoms since childhood was diagnosed with scimitar syndrome. Radiological assessments, including chest X-rays, computed tomography pulmonary angiograms (CTPA), and transthoracic echocardiography, confirmed the presence of a curved vessel originating from the right hemidiaphragm and connecting with the inferior vena cava (IVC). The patient and her medical team opted for conservative management, involving multidisciplinary care, tailored treatment for infections, and regular monitoring. The rarity of Scimitar syndrome necessitates careful diagnosis and management decisions. While surgical intervention is often recommended, this case demonstrates the complexities of choosing conservative management based on patient preferences and the evolving clinical course. A literature review reveals varied outcomes of surgical and conservative approaches, emphasizing the need for personalized strategies. Radiological techniques, such as CTPA and MRI, play pivotal roles in diagnosis and monitoring. This case report underscores the clinical significance of scimitar syndrome, particularly in regions with limited reported cases, like Pakistan. The multidisciplinary management approach, the decision-making process regarding conservative treatment, and the unique radiological findings contribute to the medical community's understanding of this rare condition.

## Introduction

Scimitar syndrome, also referred to as congenital venolobar syndrome, Halasz syndrome, mirror-image lung syndrome, hypogenetic lung syndrome, and vena cava bronchovascular syndrome, is an uncommon congenital cardiac condition. Its unique anatomical feature, resembling a curved-blade sword known as a "scimitar," gives it its name [1,2]. Although it affects only a small fraction of live births, approximately 1-3 per 100,000, it presents as a partial anomalous pulmonary venous return, causing a left-to-right shunt [3]. However, due to the potential lack of symptoms, the true incidence might be higher. Although the precise mechanism causing scimitar syndrome is not yet known, it is likely caused by an embryological mistake in the basic development of the lung bud in early embryogenesis [4].

Symptoms of scimitar syndrome encompass cyanosis, respiratory distress, tachypnea, recurrent pneumonia, and heart failure. It can be categorized into infantile and adult forms, with the former being associated with higher mortality due to severe clinical manifestations, including pulmonary hypertension and congestive heart failure [1,5]. Managing these cases necessitates a multidisciplinary approach [6]. Treatment involves redirecting systemic arteries through catheterization and surgical corrections such as tunnel baffling or re-implanting pulmonary venous drainage into the left atrium [3,7].

This case report spotlights a unique presentation of scimitar syndrome in a 26-year-old female, underscoring its clinical importance and emphasizing the need for heightened awareness among healthcare providers. Notably, this is the first documented instance of this syndrome in Pakistan, underscoring the limited prior reports within the country. By disseminating our findings, we aim to enrich the medical literature, advance comprehension of this rare condition, and ultimately facilitate more precise diagnoses and enhanced management approaches for similar cases.

## Case presentation

**Figure 1:**
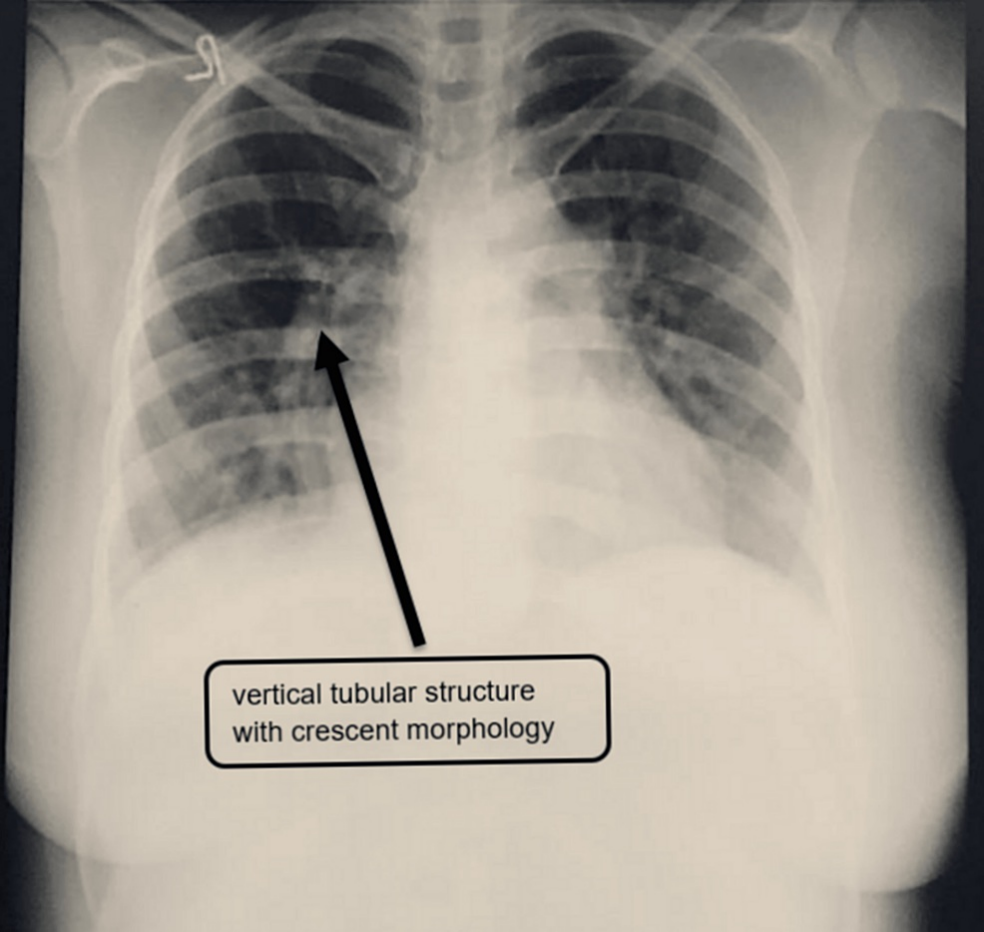
A chest X-ray shows a vertical tubular structure with crescent morphology.

A 26-year-old female patient presented with a history of recurrent pulmonary infections, fever, shortness of breath, cough, and wheezing since childhood. She had been previously diagnosed and treated for asthma, but her symptoms persisted despite treatment. The patient had been married for 12 years but had no children, and she had a history of two abortions in the past. The patient's marital history and reproductive health were also significant, with a history of two abortions and no successful pregnancies. The patient's clinical history included a long-standing pattern of respiratory symptoms, which had been attributed to asthma. However, despite appropriate treatment, her symptoms continued to worsen over the years. She reported recurrent episodes of pulmonary infections, often accompanied by fever, cough, and wheezing. The initial suspicion was raised by a chest X-ray that revealed an unusual vertical tubular structure with a crescent morphology in the right hemithorax, which can be seen in Figure 1. This was the first chest X-ray the patient ever received.

**Figure 2:**
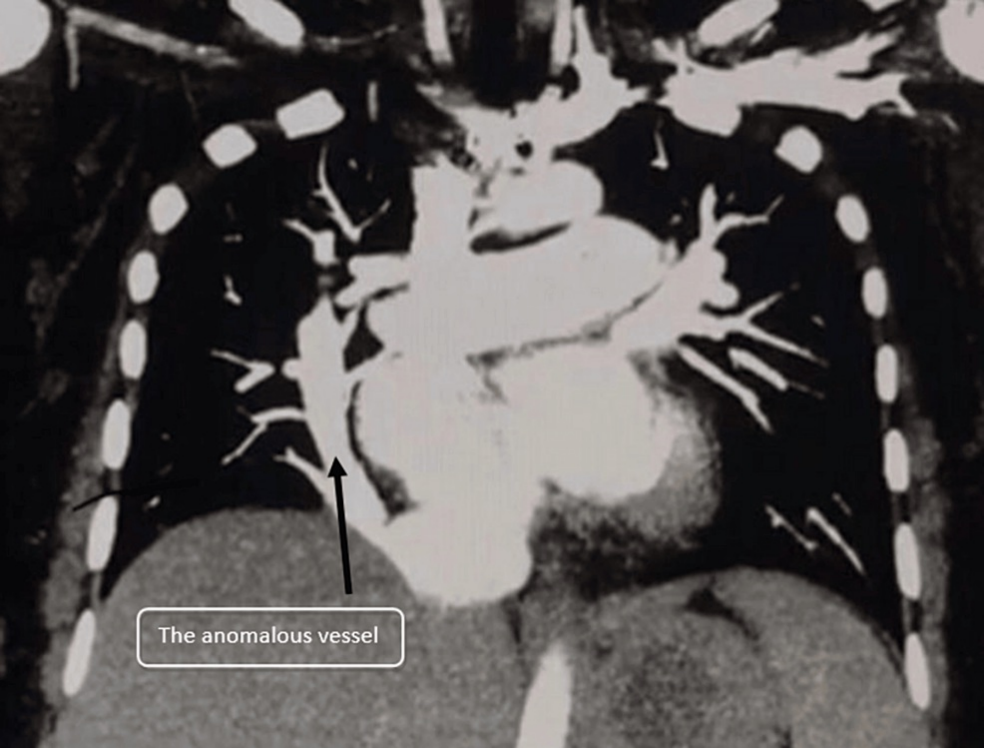
A CTPA with a peculiar vessel originating from the medial aspect of the right hemidiaphragm. CTPA: computed tomography pulmonary angiography

The chest X-ray revealed a distinctive vertically oriented tubular structure with crescent-like morphology within the right hemithorax. This discovery triggered suspicions of an underlying structural anomaly. In the subsequent steps, a computed tomography pulmonary angiogram (CTPA) was conducted to comprehensively understand the anomaly. The CTPA unveiled a peculiar vessel measuring approximately 2.1 cm that originated from the medial aspect of the right hemidiaphragm, which was a significant anomaly. This vessel was observed to connect with the inferior vena cava (IVC) just before its convergence with the right atrium. Notably, there was no noticeable loss of lung volume on either side, which strongly indicated the presence of a scimitar syndrome with partial anomalous pulmonary venous return, in line with the findings from the CT scan, as is visible in Figure 2.

Based on the radiological findings and clinical presentation, a diagnosis of scimitar syndrome with partial anomalous pulmonary venous return was established. This rare congenital heart anomaly involves the abnormal drainage of pulmonary veins into systemic veins, often resulting in a crescent-like shadow on imaging. Given the recurrent pulmonary infections and compromised pulmonary function, the option of surgical intervention was discussed with the patient.

The patient was extensively counseled about the condition, potential surgical approaches, and the associated risks and benefits. However, after thorough consideration, the patient and her medical team pursued a conservative management approach, emphasizing medical treatment, lifestyle modifications, and regular monitoring. The patient's medical management involved a multidisciplinary team, including pulmonologists, cardiologists, and other specialists as needed. She received tailored treatment for her recurrent pulmonary infections as well as ongoing management of her respiratory symptoms. Periodic imaging and clinical assessments were conducted to monitor the condition's progression and the patient's overall health.

Throughout follow-up, the patient's symptoms showed variable responses to medical management. While some exacerbations were managed effectively, there were instances where her respiratory symptoms required more intensive interventions. Despite these challenges, the patient remained committed to her conservative management approach, and her medical team continued to provide comprehensive care.

## Discussion

Scimitar syndrome, an uncommon congenital heart anomaly, involves abnormal drainage of pulmonary veins into systemic veins, resulting in distinctive crescent-like shadows in imaging. The patient's recurring pulmonary infections and compromised lung function prompted discussions regarding the feasibility of surgical intervention. However, following thorough counseling, both the patient and the medical team opted for a conservative management approach.

Gudjonsson et al. conducted a study exploring the clinical characteristics of scimitar syndrome patients. Their findings revealed a diverse range of clinical presentations in these patients, underscoring the need for individualized management strategies to achieve optimal outcomes [8]. Meanwhile, a study by Chowdhury et al. investigated the long-term impact of surgical intervention for scimitar syndrome, demonstrating its potential to alleviate clinical symptoms and enhance overall quality of life [9]. Notably, surgical correction is warranted for cases of scimitar syndrome, particularly when accompanied by atrial septal defects (ASD), pulmonary hypertension, or anomalous vein stenosis [10].

Numerous other studies have explored management strategies for scimitar syndrome. Alghamdi et al. reported that surgical intervention remains the preferred treatment strategy, especially for symptomatic patients [11]. In contrast, Dupuis et al. revealed that conservative management can effectively treat asymptomatic patients with scimitar syndrome [12].

Detecting venous anomalies within the thorax can be achieved through various approaches, including thoracic surgery, autopsy, and diagnostic procedures like chest radiography, angiography, echocardiography, computed tomography (CT), and magnetic resonance imaging (MRI). Notably, CT and MRI are highly effective in confirming irregularities in thoracic blood vessels. Contrast-enhanced computed tomography angiography (CTA) or magnetic resonance angiography (MRA) using contrast agents have gained recognition as versatile and non-invasive alternatives to traditional angiography [13,14]. Furthermore, advanced characterization of the anomalous right bronchial tree can be achieved through three-dimensional (3D) reconstructions of the tracheobronchial tree facilitated by spiral CT scans [13].

## Conclusions

This case report highlights a remarkable instance of scimitar syndrome in a 26-year-old female, shedding light on its clinical significance and underscoring the need for heightened awareness among healthcare professionals. Notably, this case represents the rarely documented occurrence of scimitar syndrome in Pakistan, emphasizing the scarcity of prior reports within the country. By disseminating these findings, the aim is to contribute to the medical literature, enhance the understanding of this rare condition, and ultimately facilitate more accurate diagnoses and improved management strategies for similar cases. The patient's complex clinical history, the unique radiological findings, and the decision-making process regarding conservative management provide valuable insights into the challenges and considerations associated with treating this condition. As such, this report serves as a valuable addition to the medical community's understanding of scimitar syndrome and its multidisciplinary management approaches.
